# Perceived Social Networks and Newborn Health: Evidence from Self-Help Group Communities in Northern India

**DOI:** 10.3390/soc8040092

**Published:** 2018-09-20

**Authors:** Jenny Ruducha, Xinran Huang, James Potter, Divya Hariharan, Danish Ahmad, Sampath Kumar, P. S. Mohanan, Avishek Hazra

**Affiliations:** 1 Braintree Global Health, Cambridge, MA 02140, USA; 2 Clinical Trial Data Services, Inc., Acton, MA 01720, USA; xhuang@clinicaltrialdata.com; 3 Department of Global Health and Population, Harvard T.H. Chan School of Public Health, Harvard University, Boston, MA 02115, USA; jpotter@g.harvard.edu; 4 Institute for Financial Management and Research, Chennai 600006, India; divya.hariharan@ifmr.ac.in; 5 Public Health Foundation of India and Indian Institute of Public Health, Gandhinagar 382042, India; danish.ahmad@phfi.org; 6 Rajiv Gandhi Mahila Vikas Pariyojana, Raebareli 229001, India; sampath97@gmail.com (S.K.); psmohanan@rgmvp.org (P.S.M.); 7 Population Council, New Delhi 110003, India; ahazra@popcouncil.org

**Keywords:** self-help groups, knowing network, perceived network, social network, community empowerment, community development, newborn health, breastfeeding, behavior change

## Abstract

The limitations of individual level interventions in changing behaviors to improve global maternal, newborn and child health have generated more interest in the patterns of social influence and decision making embedded in families, friends and communities. The purpose of this study is to expand the understanding of village dynamics in India and how first degree social and advice networks and cognitive perceptions of 185 recently delivered women (RDW) in areas with and without women’s Self-Help Groups (SHGs) affect immediate breastfeeding. Data was collected in 6 blocks and 36 villages in Uttar Pradesh, India. The expansion of RDW’s social worlds and creation of social capital through the organization of Self-Help Groups in their villages allowed us to examine basic relationships and advice formation as well as perceptions of interconnectedness of known groups. RDW living in SHG villages and blocks had consistently higher numbers of relationship ties, health advice ties and higher density of health advice networks than RDW living in the non-SHG areas. RDW’s perceived knowing ties were also significantly higher between family and health workers in the SHG areas with related higher immediate breastfeeding rates. These results suggest that SHGs can accelerate community social capital and promote more accountability in the health system to engage with families and support the change from traditional to more evidence-based health practices.

## 1. Introduction

### 1.1 Background and Study Purpose

Goals [1]. In lower income countries with low performing health systems, the actions of individuals and communities in adopting lifesaving behaviors and demanding better quality services from local providers becomes a more important pathway to consider in improving health [2]. The limitations of individual level interventions have generated more interest in the social environment and networks including the patterns of social influence and decision making embedded in families, friends and communities [3]. Decades of research demonstrate that social network interventions can have positive effects in global health programs to promote family planning methods [4], bullying reduction among adolescents [5] smoking and alcohol cessation [6–9] and reduction of sexually transmitted diseases and HIV/AIDS [10,11].

The purpose of this study is to expand the understanding of social and advice networks of recently delivered women (RDW) that constitute a critical input into health behavior of women affecting maternal and newborn health outcomes. Beyond the first-degree “knowing and advice” networks, we are also interested in the perception of women about who has relationships with whom in their community that could constitute an effective force for continuing stagnation or positive change. Our study is built on the Uttar Pradesh Community Mobilization project (UP CMP), in India, that utilizes women’s microfinance organizations of Self-Help Groups (SHGs) and their federations at village and block levels, as platforms for health message dissemination and development of linkages with the local health system of providers. The expansion of recently delivered women’s (RDW)’s social worlds through the organization of SHGs in their villages will allow us to examine this effect on basic relationship and advice formation as well as perceptions of interconnectedness of known groups. Further inroads in understanding the social networks dynamics as perceived by RDW can create knowledge and social capital that can be used to improve the development and execution of more effective health interventions and community progress.

### 1.2 Cognitive and Social Network Analysis

The continuing poor health outcomes and inequity between groups based on social and economic factors has been very difficult to overcome in many settings and especially in low-income countries [1]. The frameworks for studying and developing interventions for behavior change have been skewed towards the individual as a change agent in making decisions, as described in the health belief model, theory of reasoned action, social learning theory and the protection motivation theory [12]. Effective on a small scale, many interventions have become unsustainable when funding is removed or if programs are expanded, they often do not deliver the same results [13]. The testing of new integrated paradigms and approaches to study health behavior change is limited and this study provides an important move in that direction. We view human behavior as a complex process that is part of an integrated set of social practice theories that recognizes that human practices (ways of doing, ‘routinized behavior’, habits) are themselves arrangements of various inter-connected ‘elements’, such as physical and mental activities, norms, meanings, technology use, knowledge, which form peoples’ actions or ‘behavior’ as part of their everyday lives [14]. The last decade has led to the increased use of social network analysis and perceived cognitive social structures. This development provides an expanded view into cognitive variations in women’s perspectives of the degree of interconnectedness in different community settings that can lead to insight into the process of changing behaviors. Additionally, the global growth of microfinance options for poor women and the creation of SHG platforms can expand social networks and opportunities for influence, which could potentially drive the health of mothers, children and their communities.

Both cognitive and social network analyses (SNA) describe the patterns of interactions that define the behaviors and experiences of individuals within the social environments in which they live and work, that form the core element of social capital [15]. But whereas SNA focuses on the actual configuration of ties surrounding individuals, cognitive social structure (CSS) research describes these patterns of interactions as perceived by individuals [16]. In exploring this frontier between the cognitive and behavioral realms of networks, the focus of most CSS research has been to compare and contrast individuals’ network cognitions with the actual social network surrounding them [17] and to trace their respective influences on outcomes [18].

Despite the increasing interest in understanding the social determinants of health at the individual and broader community levels, the literature on social networks and health conducted in low and middle income countries (LMICs) is limited [2]. This also holds true for cognitive social network research [19,20] that explores how individuals perceive and cognitively represent the networks of relationships around them. The research that is available is directed at the structure of perceived cognitive networks rather than on the effects of these cognitive networks on outcomes [16]. Existing literature is centered on adolescents and risk behavior in the United States [21]. Other studies on CSS have focused on the relationship between the degree of power in social networks and the cognitive accuracy of social network perceptions [22–24]; and the relationship of CSS to loneliness [25,26].

### 1.3 Self-Help Groups in India and Role of Social Capital

In the case of India, community and village level social interactions form the bedrock of several policy and development focused interventions. SHGs are generally ‘homogenous groups’ and members tend to be from similar socio-economic backgrounds and live in close proximity to one another. These groups are engaged in collective saving and thrift activities for access credit on the basis of mutual liability between the members of the group [27]. The model not only aims to alleviate poverty but also to empower women to promote the development of their local communities and improve awareness and decision-making abilities. While the primary reason for joining these groups for women is to access credit, SHGs also contribute towards breaking traditional barriers faced by women, and enhancing levels of self-confidence, self-efficacy and self-esteem [28]. Accordingly, mobilization of women’s SHGs is understood as a critical strategy to address issues of exclusion and marginalization among communities and to promote social capital and community empowerment through the expansion of networks.

Microfinance and self-help groups have emerged as a tool for socio-economic change, and have helped communities to grow and develop, voice their concerns and increase interaction with others [29]. Encouraging and strengthening the formation of new networks among communities, especially while implementing development programs, can directly contribute towards empowerment through improving community members’ awareness and knowledge concerning community problems [30]. A recent study confirmed the power of information diffusion through SHGs by concluding that a microfinance participant is seven times as likely to transmit information to another household as a non-participant [31]. The increase in social networks and social interactions among SHGs therefore, have the potential to improve the status of women, as well as play a crucial role in the development of microfinance institutions [32]. Since our research is focused on understanding of social and advice networks and perceived networks of RDW in the context of Uttar Pradesh, India, where SHG or microfinance institutions exist, it is important to also understand the SHGs landscape, their networks and status of interaction.

## 2. Materials and Methods

### 2.1 Study Population and Data Collection

The current study is nested within the larger Uttar Pradesh Community Mobilization Project (UP CMP) in India. The main aim of the UP CMP is to utilize an existing network of federated SHGs to disseminate important messages related to Maternal and Newborn Health. These SHGs are set up in rural areas where health messages are more difficult to disseminate through traditional methods. Women who receive health messages through SHGs may also be receiving messages through a number of other sources, including their family members, community members, as well as a number of government health workers, some of whom are stationed within each village or within local catchment areas.

The data for this study came from a sample of 185 recently delivered women (RDW) from six different Districts of Uttar Pradesh, selected to capture geographic diversity within UP and diversity of exposure to SHG programming. Within each District, one Block was purposively sampled in order to portray a variety of experiences, with major criteria being the age and penetration of the SHG network in the Block and basic development indicators for each Block that were available at the time of the study. In total, four Blocks were selected with SHG activity, and two Blocks were selected in which there was no SHG activity at the time of the study. Within each Block, six villages were randomly sampled for inclusion in the study. Within each village, up to five interviews were conducted with RDW. Two RDW each (one SHG member and one non-member) were selected from the main village town and from a hamlet containing SHGs, and one additional non-SHG member RDW was selected from a hamlet without SHGs present. If more than five RDW were present in a village at the time of the study meeting inclusion criteria, then five were sampled purposively.

All RDW completed face-to-face interviews. An ego-centric network questionnaire was used to collect the relational data between index cases (egos) and fixed list of potential people in their networks (alters). Questionnaire data included demographics, health outcomes, network measures and affiliations. This analysis was restricted to the relational data between egos and their family members, SHG members and health workers. Family members include husband (H), mother (M), mother-in-law (ML), maternal household (MH), paternal household (PH) and also friend (Fr). SHG members include swasthya sakhi or health volunteer (GSS), meeting sakhi (MS), village organization swasthya sakhi (VOSS), village organization member (VOM), village organization office bearer (VOB), Rajiv Gandhi Mahila Vikas Pariyojana staff, the organization supporting SHG development (RG). Health workers include Accredited Social Health Activist (ASHA), voluntary village health worker paid for specific tasks, Auxiliary Nurse Midwife (ANM), trained and salaried government worker who runs monthly village Health and Nutrition Days, and Anganwadi Worker (AWW), a nutrition and day care focused salaried government worker. We refer to the three village based health and nutrition workers as “AAA”.

Each RDW was asked how she was connected with family, SHG members, health workers and other key community members who influenced knowledge and access to health information and related services. A series of questions were asked concerning the knowing and health advice ties between each RDW and a fixed list of 18 alters. Both of these two types of ties were unidirectional and unconfirmed. A knowing tie was said to exist between an RDW and a person in her reported network, if she acknowledged that it existed. A health advice tie was defined as when an RDW received health advice from a person in her self-reported network. The knowing ties and health advice ties constitute an RDW’s first-degree network.

**Figure 1:**
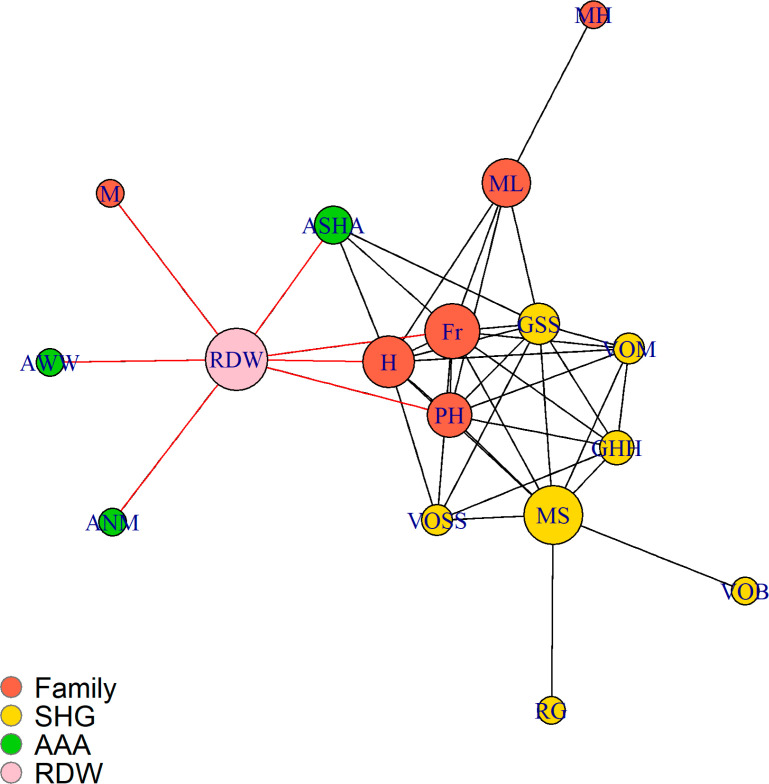
Example of a recently delivered woman’s (RDW)’s ego-centric network. Specific color nodes represent the different affiliations of the RDW with: RED nodes for Family; YELLOW nodes for SelfHelp Group (SHG) members; and GREEN nodes for village health and nutrition workers. The red ties were the health advice ties in the RDW’s first-degree network and black ties represent the perceived knowing ties, that is, who in her network she thought knew each other.

Based on the first-degree networks, each RDW was asked how she perceived the knowing ties (perceived knowing ties) between every two people in her self-reported network. For each RDW, a perceived knowing tie between person i and person j was said to exist only when both of the person i and j were in this RDW’s self-reported network and this RDW perceived that person i and j knew each other. The first-degree network and perceived network comprise an RDW’s ego-centric network (Figure 1).

In this example, the RDW receives advice from the three health workers (AAA), family members and a friend. She does not receive direct advice from the SHG members. Her cognitive perceptions of who is affiliated with whom in her social world is depicted by black ties. There was no validation of whether her perceived knowing ties actually existed or not, but studies have demonstrated that people are able to perceive, record and report accurately on who interacts with whom among the people around them [33]. The RDW organizes her perceptions according to a design or grouping structure as demonstrated in Figure 1, where family and SHG in particular are clustered together within dense sub-groups. Key members (depicted by larger nodes) constitute bridging ties that facilitate linkages with others. The AAA are perceived as not having linkages with each other and only the ASHA, the community-based worker is perceived as having relationships with the RDW’s two family members and one SHG member.

### 2.2 Measures

Demographic measures included age, literacy, education, caste and SHG membership. The SHG membership includes three categories: (1) SHG members; (2) being part of an SHG network; and (3) not being SHG members and not being part of SHG network. Being part of an SHG network refers to RDW who were not SHG members but had household members or friends or neighbors who were SHG members. The primary health outcome was immediate breastfeeding and defined as breastfeeding within one hour of delivery.

Ego-centric network measures include: knowing ties, health advice ties, perceived knowing ties and density. Density captures the proportion of all possible ties that are actually present. The density of an ego-centric network was defined as sum of the knowing ties of an RDW divided by the total number of alters. Density of health advice was calculated separately for each of the three affiliations in the RDW’s community. We defined density of health advice is sum of the health advice ties that an RDW received from an affiliation divided by sum of the knowing ties of this affiliation. Perceived knowing ties and density of perception were calculated for every two affiliations of an RDW’s self-reported network. Density of perception reflected the proportion of perceived knowing ties. This was derived by dividing the total perceived knowing ties by the total pairs of alters between two affiliations in the RDW’s self-reported network.

### 2.3 Statistical Analysis

This is a univariate exploratory analysis. All of the statistical tests were conducted on data that were not adjusted for potential confounders. Continuous statistics were described as mean and standard deviation for normal data or median and interquartile range for non-normal data. Binary statistics were shown as frequencies and proportions. Two-sample *t* test was used to compare the means between two groups. To account for non-normality of the data, Exact Wilcoxon rank sum test was used to compare the distribution between two groups. Kruskal–Wallis test was used to perform non-parametric multiple comparisons and Benjamini–Hochberg adjustment was used to control for false discovery rate in pair-wise comparison. Analysis was performed in R 3.5.0.

## 3. Results

Data were collected from 185 RDW aged between 18 and 43 years old (*M* = 25.15, *SD* = 4.46). Table 1 presents the summary statistics for the study sample. Among them, 58.4% were literate, 60% received any education, 44.9% were of scheduled caste or scheduled tribe; 19.5% of the RDW were SHG members and 27.6% were part of SHG networks, and 57.8% engaged in immediate breastfeeding. The average density of network was 0.58 (*SD* = 0.18) and there were 10.43 (*SD* = 3.24) knowing ties and 7.28 (*SD* = 4.07) health advice ties.

**Table 1:** Recently Delivered Women’s Summary Statistics.

Measures	Summary Statistics N = 185
Demographic	
Sample size	185
Age, mean (*SD*)	25.15 (4.46)
Literate, N (%)	108 (58.4)
Received any education, N (%)	111 (60.0)
Belong to scheduled caste/tribe, N (%)	83 (44.9)
Residence	
Self-Help Group (SHG) Blocks & villages, N (%)	120 (64.9)
Non-SHG Blocks & villages, N (%)	65 (35.1)
SHG membership	
SHG members, N (%)	36 (19.5)
Part of SHG network, N (%)	51 (27.6)
Not SHG members, N (%)	98 (53.0)
Heath outcome	
Immediate breastfeeding, N (%)	107 (57.8)
Network measures	
Density, mean (*SD*)	0.58 (0.18)
Knowing ties, mean (*SD*)	10.43 (3.24)
Health advice ties, mean (*SD*)	7.28 (4.07)

N: number; %: proportion; *SD*: standard deviation.

### 3.1 First-Degree Networks

RDW had 6.36 (*SD* = 0.96) knowing ties or number of connections with family, 2.46 (*SD* = 0.96) knowing ties with health workers and 2.48 (*SD* = 3.04) knowing ties with SHG members (Table 2). As there were many more potential family members to receive advice from, the average number of health advice ties of family was 4.14 and twice the health advice ties of health workers and SHG members. The density of health advice of family was 0.66 (*SD* = 0.34), significantly lower than for health workers (*M* = 0.77, *SD* = 0.38, *p* < 0.001) and SHG members (*M* = 0.76, *SD* = 0.33, *p* = 0.0179).

**Table 2:** First-Degree network measures between RDW and their affiliations, mean (*SD*).

Affiliations	Knowing Ties	Health Advice Ties	Density of Health Advice
Family	6.36 (0.96)	4.14 (2.19)	0.66 (0.34) ^a^
SHG blocks	6.41 (0.95)	4.21 (2.25)	0.66 (0.34)
Non-SHG blocks	6.26 (0.99)	4.00 (2.09)	0.65 (0.34)
Health workers	2.46 (0.96)	1.91 (1.25)	0.77 (0.38)
SHG blocks	2.63 (0.83) ^b^	2.08 (1.18) ^b^	0.79 (0.36)
Non-SHG blocks	2.17 (1.11)	1.60 (1.32)	0.73 (0.43)
SHG members	2.48 (3.04)	1.90 (2.63)	0.76 (0.33)

a The density of health advice of family was significantly lower than for health workers and SHG members;

b RDW in the SHG blocks had significantly higher knowing ties and health advice ties with health workers than RDW in the non-SHG blocks.

Among the 185 RDW, 120 had potential access to SHG members due to their residence in SHG villages within four of the six blocks under study. RDW in the SHG blocks had consistently higher knowing ties, health advice ties and density of health advice than RDW in the non-SHG blocks. Significant difference was found in the relationships between health workers (AAAs) and RDW. RDW in the SHG blocks had 0.46 more knowing ties with health workers (*p* = 0.0046; Table 2) and 0.48 more health advice ties with health workers (*p* = 0.0129) than those in the non-SHG blocks.

### Perceived Networks

Table 3 displays perceived network measures between different sub-groups. RDW in the SHG blocks were consistently higher in the potential knowing ties, perceived knowing ties and density of perception between family and health workers. The mean density of perception between family and health workers was 0.68, significantly lower than for between family and SHG members (*M* = 0.71, *p* = 0.0243) and for between SHG members and health workers (*M* = 0.77, *p* = 0.0017).

**Table 3:** Perceived network measures, mean (*SD*).

Perceived Network	Pairs of Alters	Perceived Knowing Ties	Density of Perception
Family-Health workers	16.30 (4.97)	11.02 (5.48)	0.68 (0.27) ^a^
SHG blocks	17.30 (4.53)	11.94 (5.16)	0.70 (0.25)
Non-SHG blocks	14.14 (5.18)	9.16 (5.69)	0.64 (0.30)
Family-SHG members	27.37 (19.09)	18.40 (14.53)	0.71 (0.28)
SHG members-Health workers	12.43 (8.51)	9.16 (2.63)	0.77 (0.35)

a The density of perception between family and health workers was significantly lower than between family and SHG members and between SHG members and health workers.

**Figure 2:**
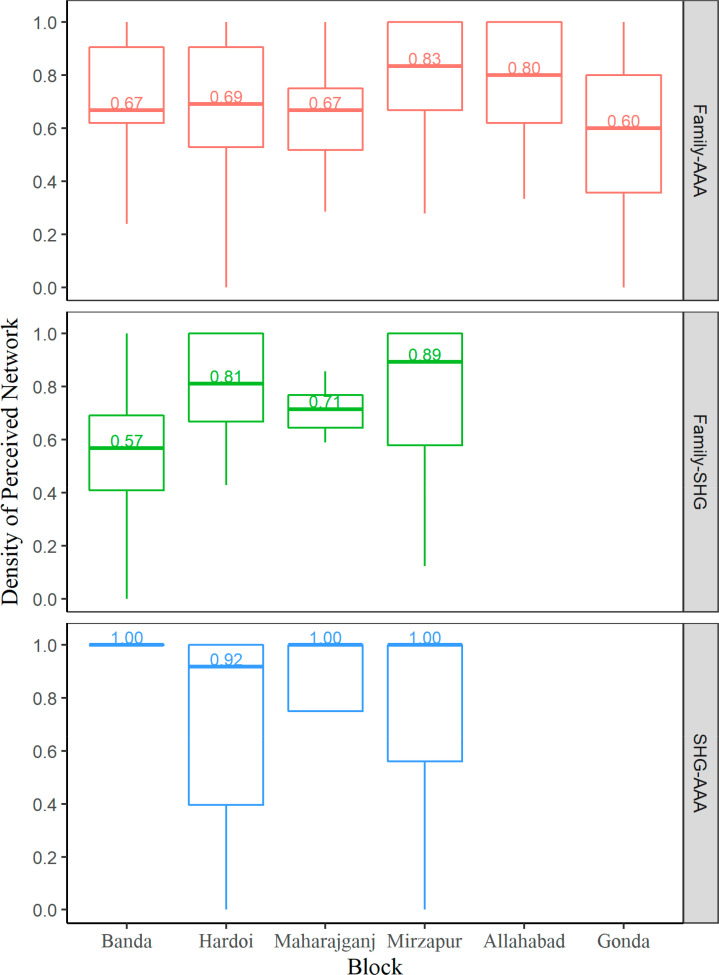
Density of perception by block and perceived network. The density of perception between family–three health workers (AAA), family–SHG and SHG–AAA are represented by red, green and blue boxplots, respectively. Figures within each box are the median density of perception. Please note that that the first four blocks have SHG engagement (Banda, Hardoi, Maharajganj and Mirzapur) whereas Allahabad and Gonda did not have SHGs, hence those relationships involving SHGs are left blank.

Among the six blocks, Allahabad, a non-SHG area, was similar to Gonda, another non-SHG area, in the potential knowing ties between family and health workers but was significantly lower than the four SHG areas of Mirzapur (*p* = 0.0043), Maharajganj (*p* = 0.0015), Banda (*p* = 0.0071) and Hardoi (*p* = 0.0002). Further examination revealed that Allahabad had the lowest knowing ties of ASHA, a type of health workers, among the six blocks (*p* = 0.0001; Table 4) while Gonda was not significantly different from the rest of the five blocks. Figure 2 displays the density of perception between family and health workers by six blocks. Gonda had the least density of perception (*Mdn* = 0.60, IQR = 0.36–0.80) and was significantly lower than Mirzapur (*Mdn* = 0.83, IQR = 0.67–1.00, *p* = 0.0038) and Allahabad (*Mdn* = 0.80, IQR= 0.62–1.00, *p* = 0.0164). Maharajganj was also significantly lower in the density of perception than Mirzapur (*p* = 0.0210). Regarding the block-wise perception involving SHG members, due to large variability within each block, no difference was observed, except that Banda had a significantly lower density of perception between family and SHG members than block Hardoi (*Mdn* = 0.57, IQR= 0.41–0.69 vs. *Mdn* = 0.81, IQR = 0.67–1.00, *p* = 0.0194).

**Table 4:** Distribution of Accredited Social Health Activist (ASHA)/Auxiliary Nurse Midwife (ANM) knowing ties among blocks.

Affiliations	Blocks	Knowing Ties, N (%)
ASHA	SHG blocks Banda Hardoi Maharajganj Mirzapur	20 (74.1) 28 (87.5) 27 (90.0) 22 (71.0)
	Non-SHG blocks Allahabad	14 (41.2) ^a^
	Gonda	23 (74.2)
ANM	SHG blocks Banda Hardoi Maharajganj Mirzapur	(74.1) (65.6) (73.3) 19 (61.3)
	Non-SHG blocks Allahabad	15 (44.1)
	Gonda	21 (67.7)

a RDW in the Allahabad had the lowest knowing ties with ASHA among the six blocks (*p* < 0.001).

Among the upper 50th percentile of subjects residing in the four SHG blocks, the density of perception between SHG members and health workers was the highest and least variant across blocks. Gonda had the least density of perception between family and health workers, significantly lower than Mirzapur and Allahabad. Maharajganj was significantly lower in the density of perception between family and health workers than Mirzapur. Banda had significantly lower density of perception between family and SHG members than block Hardoi.

### 3.3 Application to Breast Feeding

RDW in the SHG blocks had significantly higher proportion of immediate breastfeeding than those in the non-SHG blocks (66.7% vs. 41.5%, *p* = 0.0010). Gonda, a non-SHG block, had the lowest proportion of immediate breastfeeding among six blocks (32.3%). See Table 5.

**Table 5:** Proportions of immediate breastfeeding among blocks.

Blocks	Immediate Breastfeeding, N (%)
SHG blocks	80 (66.7) ^a^
Banda	17 (63.0)
Hardoi	18 (56.3)
Maharajganj	18 (60.0)
Mirzapur	27 (87.1)
Non-SHG blocks	27 (41.5)
Allahabad	17 (50.0)
Gonda	10 (32.3) ^b^

a RDW in the SHG blocks had significantly higher proportion of immediate breastfeeding than the non-SHG blocks;

b Gonda had the lowest proportion of immediate breastfeeding among six blocks.

**Figure 3:**
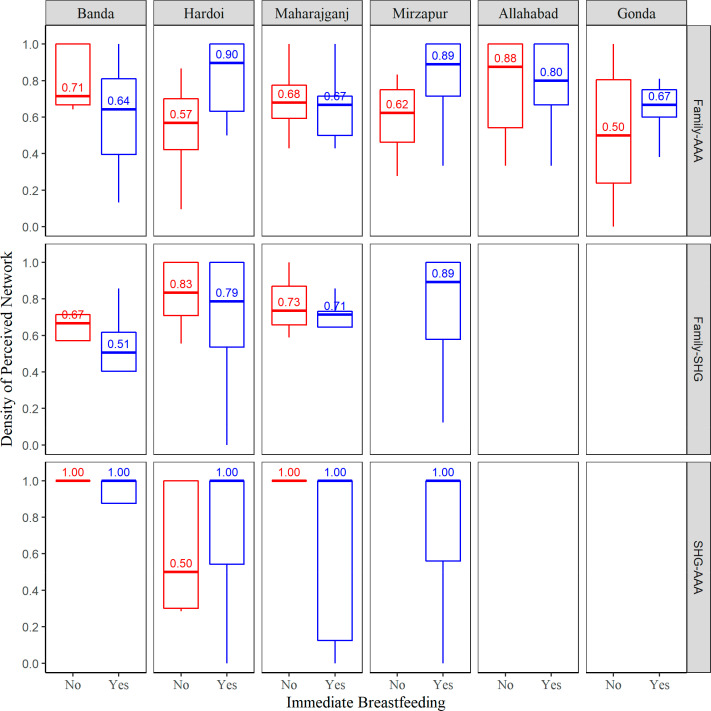
Density of perception by block, perceived network and immediate breastfeeding. Red boxplots represent non-immediate breastfeeding group and blue boxplots represent immediate breastfeeding group. Allahabad and Gonda were non-SHG blocks hence those relationships involving SHGs are left blank.

Figure 3 displays the density of perception by perceived network, block and immediate breastfeeding. Among the upper 50 percentiles of RDW residing in the four SHG blocks, the density of perception between SHG members and health workers was the highest and least variant across blocks. Mostly all the RDW perceived that all the SHG members and health workers knew each other whether they did or did not breastfeed. The only exception is Hardoi, where approximately half of RDW’s who did not breastfeed immediately, also did not perceive that SHG members and health workers knew each other. Overall, those who reported immediate breastfeeding had significantly higher density of perception between family and health workers compared to those who did not breastfeed (*M* = 0.72, *SD* = 0.25 vs. background and Study Purpose *M* = 0.63, *SD* = 0.28, *p* = 0.0344). However, due to large variability in both of the breastfeeding groups within each block, significant difference in the density of family-AAA perception between breastfeeding groups was only observed in Hardoi. RDW in Hardoi who reported immediate breastfeeding had a significantly higher density of Family-AAA perception compared to those who did not breastfeed (*Mdn* = 0.90, IQR = 0.64–1.00 vs *Mdn* = 0.57, IQR = 0.42–0.70; *p* = 0.0072). In both of the perception measures between family-SHG and SHG-AAA, no difference was observed between breastfeeding groups. In Mirzapur, RDW that immediately breastfed perceived SHG linkages both with family and AAAs. The density of perception between family and health workers was 0.75 (*SD* = 0.31) and the density of perception between SHG members and health workers was 0.73 *(SD* = 0.36).

## 4. Discussion

The purpose of this study was to explore how first degree social and advice networks and cognitive perceptions of RDW in areas with and without the activation of SHGs affect community relationships and newborn outcomes. We observed that RDW living in SHG villages and blocks had a consistently higher relationship or knowing ties, health advice ties and higher density of health advice networks than RDW living in the non-SHG areas. RDW’s perceived knowing ties were also significantly higher between family and health workers in the SHG areas with significantly higher immediate breastfeeding rates in those areas. SHGs may serve as bridging social capital that improved RDW’s access to health messages and increased the linkages of the local health system of providers with family members of RDW. These patterns are in line with several previous studies suggesting that bridging social capital has a positive effect on health [34,35].

This study shows that RDW in SHG Blocks have significantly higher perceived interactions between family and AAA. The addition of groups beyond family groups into the cognitive perceptions of RDW’s can increase exposure and legitimacy of new health behaviors as family carry traditional practices as compared to health workers or AAAs who encourage adoption of modern practices such as immediate breastfeeding. Generally, communication is most likely and effective within homophilous or similar social networks where family and community groups share a common understanding based on their cultural environment. However, homophily can inhibit people from changing their perspectives. The Diffusion of Innovations (DoI) theory asserts that homophily can ‘act as a barrier to the flow of innovations in a system’ [36] and that some heterophily is therefore essential for diffusion of innovation to occur. Ideally, ‘the individuals would be homophilous on all other variables—even though they are heterophilous regarding the innovation’ [36]. The role of the SHG platform fits well into the DOI model, as women in SHGs have the same cultural and social characteristics as the RDW yet they learn new health behaviors and actions to improve linkages with the health system. The creation of more community social capital translates into the expansion of linkages and perceived connectivity between family and health workers that further legitimize the adoption of new health behaviors.

However, the higher density and degree centrality of RDW’s perceived relationships between SHGs and AAAs does not necessarily translate into the engagement of immediate breastfeeding when individual blocks are examined. Our interpretation of this finding is subject to the sample size limitations of the study, characteristics of SHG’s and their evolving level of knowledge and health education of RDW and the village community on breastfeeding. SHGs may not have yet developed firm convictions for behavior change in their communities, especially when their ideas may be in direct conflict with accepted social norms that they may not be willing to challenge. Going back to the homophily principle, people with similar characteristics associate with each other and have similar attitudes and beliefs that reinforce traditional practices. The cultural dynamics are complicated as the ASHA health worker and Anganwadi nutrition worker (part of the AAAs) may be similar to SHG members and RDW but the ANM is more highly educated and is usually not a local resident. Given the fact that there are studies that support the notion that conflicting attitudes can even exist in similar social networks [37] and beliefs may be poorly correlated across different issues [38], the lack of consistency and adoption of new behaviors across different sub-groups may represent this dual tension. People are reasonably accurate in reporting their friends’ views about certain matters, but they may not have the level of issue specific awareness about where their known groups of relationships stand. Hence the strength of perceiving important linkages in the RDW’s social world may be less powerful if communication is lacking between any of the network members.

This study has some limitations. First, the differences we found between SHG and non-SHG blocks may not be generalized to other populations. We conducted an exploratory analysis with descriptive results that do not infer causality. We conducted analyses on data that were unadjusted for potential confounders. Due to relatively small sample size, we were unable to take into account the between-block variability and within-block variability such as age, education and caste. The small sample size also resulted in a low level of power for significance testing among blocks. Notwithstanding these shortcomings, this study helps to expand the understanding of village dynamics in India, making a unique contribution to the understanding of the association between perceived networks and breastfeeding, a contributing variable in reducing neonatal mortality. However, further studies are needed to unpack the different routes and network formations that directly and indirectly influence behavior change in different communities in the process of transition from traditional to more modern ways of living.

SHGs have demonstrated the capacity to positively impact economic well-being [39] and a range of health outcomes among women and their children [40–42]. In the study blocks, the RDW were able to challenge existing socio-cultural norms associated with breastfeeding practices through the injection of SHGs that expanded the relationships between different groups, especially RDW families and health workers. These findings contribute to the multidisciplinary research of how actual and perceived cognitive networks are affected by the infusion of social capital created by SHGs and the microfinance movement. While empowerment indicators were not separately assessed, the changes in health behaviors suggest that the existence of SHGs can lead to increased social capital with the ability to influence networks and network perceptions leading to changes in community behaviors.
